# Dysplasia epiphysealis hemimelica of the radial head: a rare case report

**DOI:** 10.1186/s12891-021-04004-2

**Published:** 2021-02-05

**Authors:** Hiva Mohamadian, Mansour Bahardoust, Borzouyeh Molazem Sanandaji, Saba Saberi

**Affiliations:** 1 Rasoul-e-Akram Hospital, Iran University of Medical Sciences, Tehran, Iran; 2 Department of Epidemiology, School of Public Health, Shahid Beheshti University of Medical Sciences, Tehran, Iran; 3 Bone and Joint Reconstruction Research Center, Shafa Orthopedic Hospital, Iran University of Medical Sciences, Tehran, Iran

**Keywords:** Dysplasia epiphysealis hemimelica, Trevor’s disease, Radial head, Elbow

## Abstract

**Background:**

Dysplasia epiphysealis hemimelica (DEH) is a rare benign overgrowth generally affecting the epiphyses and short bones of the lower limbs. DEH in the elbow joint is extremely rare, and to date, only three cases of DEH have been reported in the radial head.

**Case presentation:**

In this study, we report a case of DEH located in the radial head of the right elbow of a 10-year-old boy, which was presented with elbow pain and limited range of motion. In clinical examination, an asymmetrical enlargement was observed over the elbow. The lesion was resected surgically, and the patient’s symptoms resolved afterward. The histologic analysis of the lesion confirmed the diagnosis of DEH.

**Conclusion:**

This report highlights the role of DEH in the differential diagnosis of elbow pathologies, particularly its differentiation from osteochondroma.

## Background

Dysplasia epiphysealis hemimelica (DEH), also known as Trevor’s disease, is a nonhereditary developmental disorder with unknown etiology consisting of a benign intra-articular mass confined by epiphysis of long bones [1, 2]. It is an osteocartilaginous lesion resulting from an abnormal proliferation of cartilage tissue, which generally involves one-half of the epiphysis, therefore termed ‘hemimelic’ [2]. DEH is an extremely rare condition that mainly affects children aged 2–15 years, with an incidence of 1 in 1,000,000 individuals [1, 3]. Although the histological findings of DEH are similar to osteochondroma, its clinical, radiological, histological, and molecular characteristics favor a different entity [4]. However, differentiation of DEH with chondroblastoma [5] and parosteal osteosarcoma could be challenging, particularly in the early stages [6].

DEH mostly occur in the lower limb, and upper limb involvement is very rare. The carpal bones and the wrist are the most frequent upper-extremity involvement of DEH [7]. To the best of our knowledge, only a few numbers of DEH have been reported around the elbow [8]. In this study, we report a case of DEH located in the radial head of a 10-year-old boy, which was presented with pain and limited elbow range of motion.

The patient’s parents provided written informed consent to use the data attributed to this case for publication.

## Case presentation

A 10-year-old boy was referred to our center with a painful right elbow. The patient and his family had no considerable medical history. The patient had no history of elbow trauma and surgery, as well. In clinical examination, an asymmetric enlargement was noticed on the right elbow. In physical examination, the elbow range of motion was restricted so that a flexion-extension motion arc of 30–90° and 15° of pronation to 20° of supination was recorded.

**Fig. 1:**
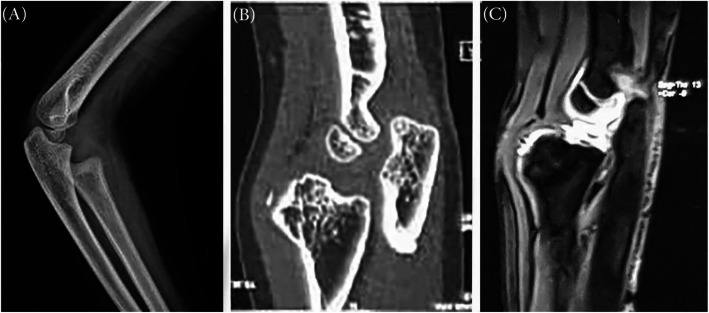
**a** Plain radiograph and **b** CT scan of the elbow showing irregular mass with focal ossification at the epiphyseal section of the proximal radius; **c** MRI of the elbow showing an epiphyseal osteocartilaginous lesion originating in the radial head epiphysis, Fig. 2: The histologic of the excised lesion revealed clusters of chondrocyte arranged in a fibrillary matrix, a thick cartilaginous cap, and ossification centers

The findings on the plain radiograph (Fig. 1a) and computed tomography (CT) scan (Fig. 1b) revealed an irregular mass with focal ossification at the epiphyseal section of the proximal radius. Magnetic resonance imaging (MRI) demonstrated an epiphyseal osteocartilaginous lesion originating in the radial head epiphysis (Fig. 1c). The annular ligament could not be seen clearly in preoperative MRI. With suspecting a diagnosis of DEH, the decision was made for resecting the lesion surgically. Under general anesthesia, in a supine position, and through the anterior-lateral approach to the radial head, the lesion was resected and sent to the pathology department for further evaluation. The remnant of the annular ligament was seen intraoperatively and resected.

**Fig. 2:**
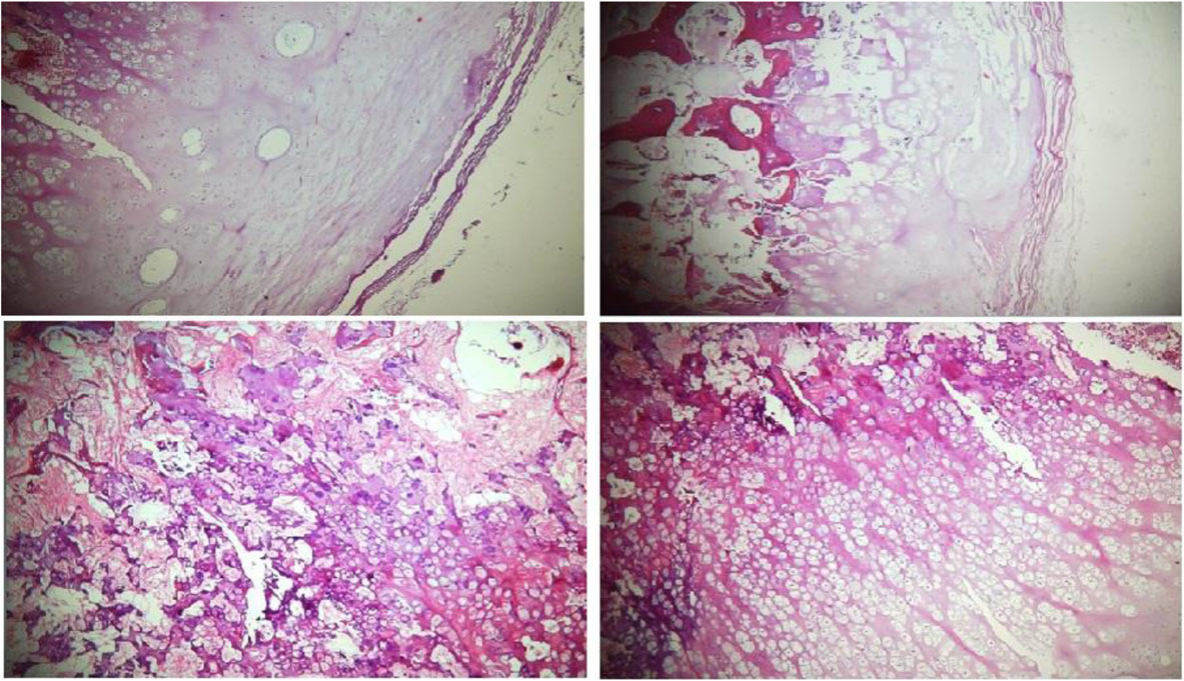
Histologic analysis of the lesion showing clusters of chondrocyte arranged in a fibrillary matrix, a thick cartilaginous cap, and ossification centers

The histologic evaluation of the excised lesion revealed clusters of chondrocyte arranged in a fibrillary matrix, a thick cartilaginous cap, and ossification centers, which were consistent with the diagnosis of DEH (Fig. 2).

**Fig. 3:**
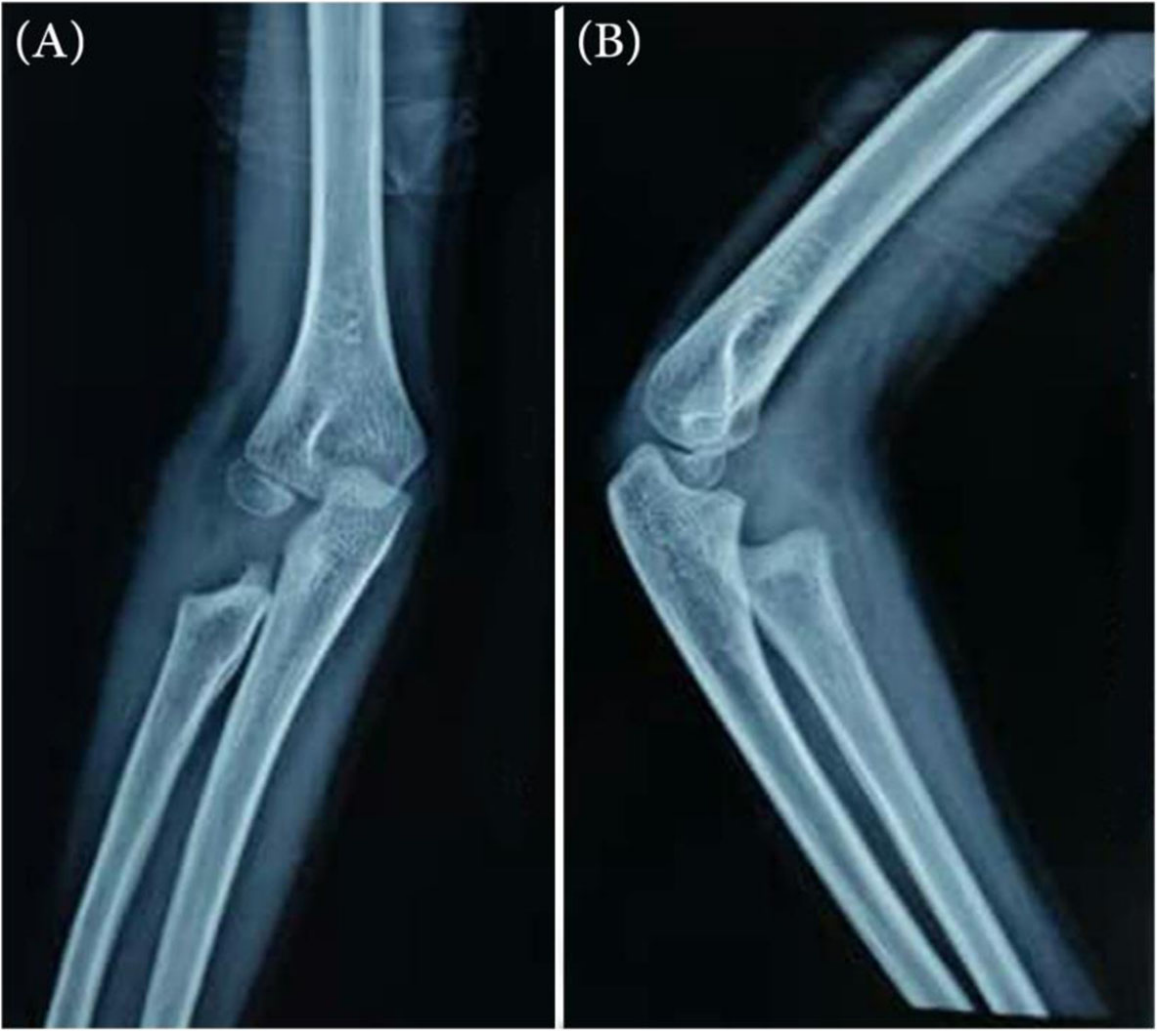
**a** Anteroposterior and **b** lateral radiograph of the elbow 1 year after the surgery showing no sign of secondary ossification or growth arrest of the forearm bone

The patient’s arm was supported in a swathe and sling for 2 weeks. The full range of motion exercises and physical therapy was administered two times a week. The first visit to the patient was 2 weeks after the surgery. The patient had no pain, and the elbow range of motion was near to normal (flexion-extension: 0–130°, pronation: 50°, supination: 50°). At the six-month follow-up, the patient was still pain-free and had a full range of elbow movements. No sign of recurrence was noticed in the follow-up radiographs. Secondary ossification or growth arrest of the forearm bones was not seen either (Fig. 3).

## Discussion and conclusion

In this study, we reported a case of DEH presented in the right elbow of a 10-year-old boy, which was associated with pain and limited elbow range of motion. The definitive diagnosis of DEH was made based on the histologic analysis of the excised specimen. The patient remained symptom-free after the resection, and no sign of recurrent was seen in the follow-up radiographs.

DEH of the upper extremity is rare. Nearly 39 cases of upper-extremity DEH have been reported in earlier literature from which DEH of the elbow is reported in 8 cases. Radial head involvement has been reported in 3 out of these 7 cases [9].

Kircher et al. reported a case of DEH in a 17-year-old boy who was admitted with the diagnosis of a recurrent cartilaginous exostosis at the radial head 4 years after initial excision. The patient was presented with elbow tenderness and limited range of motion. On the clinical examination, a progressive enlargement was noticed at the left elbow. The lesion was resected surgically, and the histological analysis of the extracted specimen confirmed the diagnosis of DEH. At the final follow-up, 16 months after the surgery, the patient was pain-free function and had no limitation in the elbow range of motion. Moreover, no sign of recurrence was presented on the follow-up radiographs [9]. The presentation and outcome of DEH in our case were very similar to the case of Kircher et al. The DEH of the elbow has also been reported in two other investigations [3, 10].

Osteochondroma is the most frequent differential diagnosis of DEH, as malignant transformation is seen in 1% of solitary osteochondromas, but not in DEH [11]. This differentiation can be made using clinical, radiologic, and pathologic parameters. While DEH generally occurs in young children aged 2–15 years and originates in the epiphysis of long bones, osteochondroma most frequently occurs at the age of 10–30 years of age and originates from the metaphysis. Histologically, DEH presents with a thick disorganized cartilage cap, whereas osteochondromas present with organized cartilage resembling the normal growth plate [4, 12].

Parosteoal osteosarcoma and DEH can also be sometimes difficult to differentiate, particularly in the early stages and if the talus is affected. In these cases, CT scanning could be very helpful in identifying calcification or ossification within the DEH lesion [13].

The decision for surgical treatment of DEH depends on several factors, such as the symptoms, location, and size of the lesion. Generally, surgical excision is only considered for those patients with pain or functional limitation. Since the malignant transformation of DEH has not been reported, observation might be enough for asymptomatic cases. However, delayed diagnosis or treatment might result in an enlarged intra-articular mass, making the decision for surgical treatment more difficult. Therefore, early excision is recommended depending on the location of DEH [14]. To avoid unnecessary surgical intervention, a preoperative biopsy could be useful, particularly in the early stages of the disease or when the lesion is presented in unusual locations [15].

Based on our experience, DEH of the radial head could be favorably treated with surgical excision, and the patient’s symptoms will completely resolve afterward. The present case raises awareness regarding the importance of DEH in the differential diagnosis of elbow pathologies, particularly with osteochondroma.

## Data Availability

Not applicable.

## Abbreviations

DEH: Dysplasia Epiphysealis Hemimelica
CT: Computerized Tomographic
